# Rediscovery of the lost skink *Proscelotes aenea* and implications for conservation

**DOI:** 10.1038/s41598-023-38286-4

**Published:** 2023-07-12

**Authors:** Ali Puruleia, Cristóvão Nanvonamuquitxo, Milagre Ernesto, Abdurabe Jamal, Iassine Amade, Wilson Monia, Yasalde Massingue, Luke Verburgt, Søren Faurby, Alexandre Antonelli, Allison Perrigo, Harith Farooq

**Affiliations:** 1 Faculty of Natural Sciences, Lúrio University, Pemba, Cabo Delgado Mozambique; 2 Enviro-Insight CC, Unit 8 Oppidraai Office Park, Pretoria, 0050 South Africa; 3 Department of Zoology and Entomology, University of Pretoria, Pretoria, 0001 South Africa; 4 Department of Biological and Environmental Sciences, University of Gothenburg, Gothenburg, Sweden; 5 Gothenburg Global Biodiversity Centre, Gothenburg, Sweden; 6 Royal Botanic Gardens, Kew, Richmond, UK; 7 Department of Biology, University of Oxford, Oxford, UK; 8 Botanical Garden, Lund University, Lund, Sweden; 9 Center for Macroecology, Evolution and Climate, Globe Institute, University of Copenhagen, Copenhagen, Denmark

**Keywords:** Zoology, Herpetology

## Abstract

Biodiversity loss is recognized as a grand challenge of the twenty-first century but ascertaining when a species is “lost” can be incredibly difficult—since the absence of evidence is not evidence of absence. This may be a relatively easy task for large and conspicuous animals, but extremely difficult for those living hidden lives or at low population sizes. We showcase this challenge by focusing on Africa’s montane skink, *Proscelotes aenea* (Barbour & Loveridge 1928). In this study, we embarked on a year-long intensive survey to find this fossorial species in Lumbo, Northern Mozambique, the only remaining location where it may still occur but was recorded for the last time over 100 years ago. We located the species already after 20 days of intensive and targeted searching by five members of our team. The finding allowed us to describe, for the first time, details on the biology and ecology of the species, alongside photos and videos of live specimens (including a pregnant female), and to sequence DNA from the species, which we used to infer the phylogenetic placement. Our combined 12S and 16S phylogenetic analysis weakly suggest that the genus *Proscelotes* may not be monophyletic and therefore requires further phylogenetic work and potentially taxonomic revision. We also gathered evidence of a possible decrease in population abundance and, based on the species' ecology, we identified urbanization as a potential key threat, which could lead to the local or global extirpation of the species. We call for urgent conservation actions that help protect the future of the montane skink, and additional surveys to map its full distribution. As countries now work towards implementing the goals and targets of the Kunming-Montreal Global Biodiversity Framework, our study demonstrates the need for proper investments in biodiversity inventories and monitoring in order to halt species extinctions by 2030.

## Introduction

In December 2022, nearly 200 nations agreed on a set of ambitious goals and targets included in the Convention on Biological Diversity’s Kunming-Montreal Global Biodiversity Framework. One of the commitments set by the Framework is that societies will, until 2030, *“halt human-induced extinction of known threatened species and for the recovery and conservation of species, in particular threatened species, to significantly reduce extinction risk”.*

While laudable and necessary, achieving such an outcome will critically depend on the ability of researchers and conservationists to ascertain the extinction risk status of all species, including the detection of species not reported for a long time and therefore potentially extinct (in which case they no longer require conservation resources). This may be a relatively easy task for large and conspicuous animals, but extremely difficult for those living hidden lives or occurring at low population densities.

One species that exemplifies this challenge well is the African montane skink, *Proscelotes aenea* Barbour & Loveridge, 1928. This is a fossorial lizard found by the naturalist Arthur Loveridge in 1918 in Northern Mozambique while clearing the land of tree stumps for the construction of the British campsite in Lumbo^1^. The species was also recorded 220 km north of Lumbo, in Pemba in 1948, and as in Lumbo, the species was never recorded there again^2^. The city of Pemba, unlike Lumbo, was extensively surveyed for herpetofauna by our research group using a wide variety of collection methods over different times of the year^3^, and it is therefore unlikely that *P. aenea* persists in or around the city.

The genus *Proscelotes* includes three species, all endemic to south-eastern Africa. *P. eggeli* is endemic to the Eastern Arc Mountains of Tanzania^4^ while *P. arnoldi* is known from two disjunct populations, one on the eastern escarpment of Zimbabwe and the other on Mount Mulanje in southern Malawi^5–7^. Unlike the other species in the genus, mostly found in montane regions, the few recorded specimens of *P. aenea* are from flat, coastal peninsulas in Mozambique, but lack further information on their distribution, biology, and ecology. This lack of information led to a Data Deficient (DD) category designation on the IUCN Red list in 2019^8^.

After its scientific description over 100 years ago, *P. aenea* has not been recorded in Lumbo again. However, only one documented expedition was made during this 100-year period, with only a very limited sampling effort, carried out by Broadley and Blake in 1965^9^. This pattern of very low or entirely absent collection efforts in remote sites is typical across Africa and is expected to be a contributing factor to the vast under-reporting of the continent’s biodiversity in international databases^10^. Because *P. aenea* was reported from Lumbo previously, and due to the low sampling effort since this initial collection, we identified Lumbo as a candidate site with a high probability of detecting and documenting *P. aenea* for the first time in many decades.

In this study, we aimed to capture individuals of *P. aenea* and to properly document the species’ biology and ecology to provide a more informed assessment of the species’ extinction risk. To do this, we conducted an intensive year-long survey in Lumbo, Mozambique with daily surveys using four different collection methods particularly suitable for the detection of this species and to cover as much seasonal and habitat variation as possible. We report the first known collections of *P. aenea* in Lumbo in over 100 years and provide supporting information on the habitat, ecology and biology of the species, including observations on its reproduction.

## Results

### Characterisation of the collected specimens

In total, eight specimens of *P. aenea* were captured over a period of 365 consecutive sampling-days. We sampled five sites but only recorded *P. aenea* in three of them (Table 1). We collected: three adult males, a pregnant female, and four juveniles (Table 1). The first record was collected on May 1st, 20 days after the beginning of the survey. Of the nine collected specimens, five were collected in the pitfalls of the trapping systems, one was born in captivity and three were collected during active search. Of the latter three, one was found inside a termite mound, one while sieving the soil with a hand rake and one on the surface of leaf litter (Table 1). Habitat photos of where the *P. aenea* specimens were found are supplied in the supplementary materials (Figure S3).

**Table 1:** IDs (field catalogue numbers), measurements (in millimetres, mm), life stage, sex, collection locality coordinates, collection method, and collection date of all *P. aenea* specimens recorded from Lumbo during the survey.

ID	STL (mm)	SCL (mm)	TL (mm)	Stage	Sex	Coordinates	Method	Date
EOS20	90	50	40	Adult	–	− 15.03°, 40.67°	PF	1/5/21
EOS144	107	62	45	Adult	M	− 15.01°, 40.66°	PF	22/8/21
EOS145	80	61	19*	Adult	M	− 15.01°, 40.66°	PF	23/8/21
EOS157	#	#	#	Juvenile	–	− 15.05°, 40.64°	AS	24/10/21
EOS173	110	70	40	Adult	F	− 15.03°, 40.67°	AS	25/9/21
EOS174	46	28	18	Neonate		− 15.03°, 40.67°	^a^	27/10/21
EOS175	49.3	30	19.3	Juvenile	–	− 15.03°, 40.67°	PF	27/9/21
EOS220	38	23	15	Juvenile	M	− 15.03°, 40.67°	PF	12/10/21
EOS269	40.6*	40.6	*	Adult	–	− 15.01°, 40.66°	AS	11/1/22

STL, Snout-to-tail length; SCL, Snout-to-cloaca length; TL, Tail-length; –, sex not determined; #, no measurements taken; PF, pitfall; AS, active search.

*Broken tail/ # damaged specimen/ ^a^ born in captivity.

**Figure 1:**
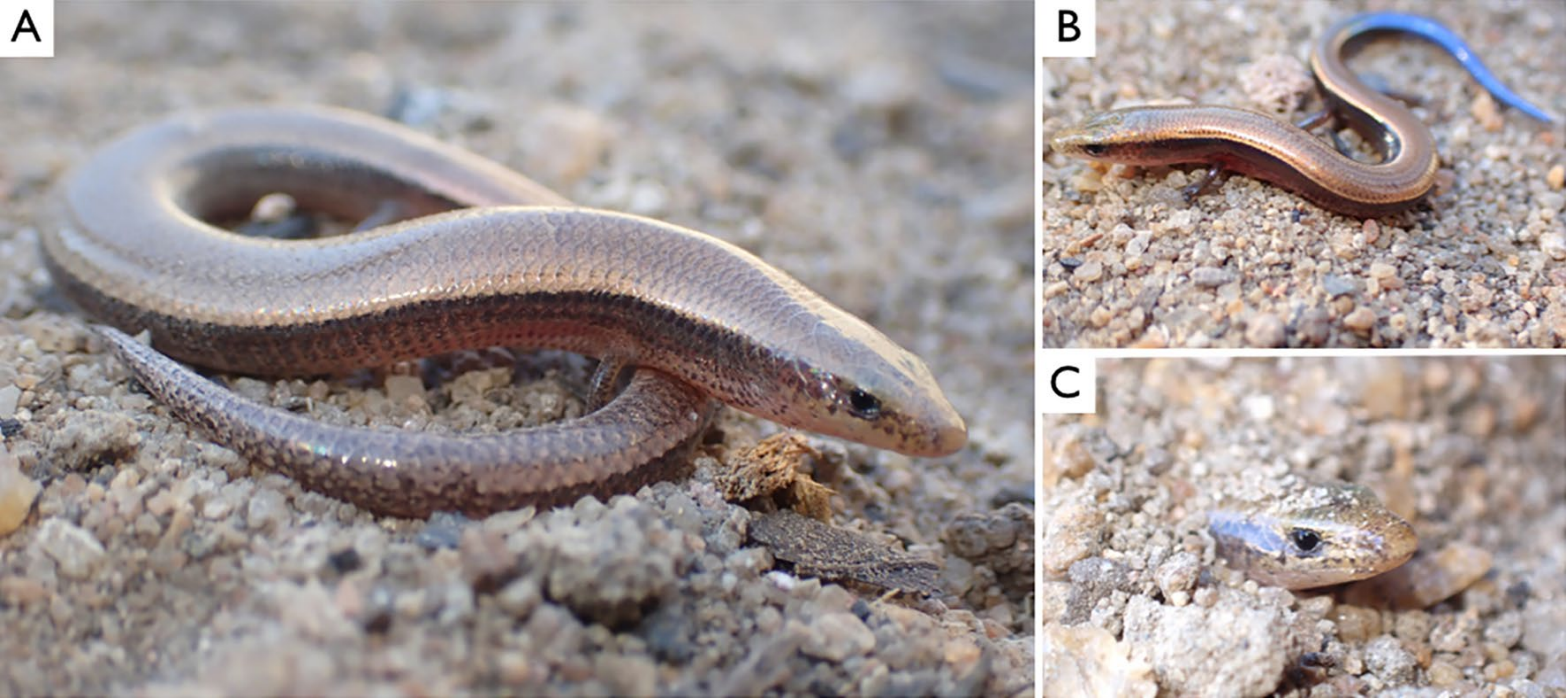
First live-specimen photographs of the montane skink, *P. aenea*. (**A**) An adult female, (**B**) A neonate and (**C**) The species hiding in loose soil.

P. aenea *morphology*: Cylindrical body, slightly flattened with anterior limbs less developed than posterior. Each limb has five digits, two of them more than twice as long as the others (the second and third digits of the anterior legs). The snout is slightly conical and projected downwards. Eyelids present. The tail length is more than half of the body length. *Scales*: Two supranasals, one frontonasal, no prefrontal, one frontal twice the length of frontonasal, six to seven lower labials, six labials with the fifth touching the eye. It has four supraoculars, six supraciliaries, two preoculars and one postocular (Fig. 1A). Without frontoparietal and has one interparietal in a triangular shape with a pineal eye, and two parietals with obtuse angulation. *Colour*: Dark spots along the head that extend until the neck in a shape of a bronze-golden ring. The venter is white with well-defined brownish or dark flanks. The adults have a tail with plumbous or grey interspersed brown spots, while juveniles are born with bright blue tails (Fig. 1B). We compared 15 anatomical structures from specimens we collected in Lumbo with the description of the ones collected in 1918. Thirteen features matched, while two (infralabials and parietal) were not mentioned in the description and were not possible to discern from the photos of the collection on GBIF. A table comparing the specimens we collected in Lumbo with the ones collected in 1918 is provided in the supplementary materials (Table S2).

### Habitat

All eight specimens of *P. aenea* were found in coastal savanna habitats, with leaf litter on sandy soil (Fig S3). The surrounding flora was composed primarily of exotic cashew (*Anacardium ocidentale*) and mango trees (*Mangifera indica*) and native Fabaceae species (e.g. *Millettia stuhlmannii)* dominated the bush community while grasses (Poaceae; e.g. *Eustachys paspaloides)* and sedges (Cyperaceae; e.g. *Cyperus polystachyos*, *Indigofera astragalina*, and *Trumfetta pantandra*) dominated the open areas. One individual was found inside a termite mound (*Cubitermes sp*).

### Locomotion, feeding and reproduction

Locomotion: The species moves with ease on both the soil surface and underground. When moving underground, individuals will frequently protrude their heads through the sand (Fig. 1C). Feeding: The specimens kept in captivity only fed on termites (*Cubitermes sp* and *Macrotermes sp.*). Usually, adults ate between 11 and 15 termites a day, but the pregnant individual ate between 26 and 71. The juveniles ate between 7 and 9 termites a day. Two captured specimens were euthanized directly, and their stomach contents were investigated, which showed a recent diet of termites. Reproduction: One specimen was pregnant and was kept alive until it gave birth (32 days after collection, ovoviviparous/viviparous). The individual gave birth to a single live young underground that measured 28 mm from snout to vent and with a blue tail of 18 mm (Table 1). Videos of the species digging, and feeding can be found on the project's website (www.extinctorshy.org).

### Phylogenetic position

**Figure 2:**
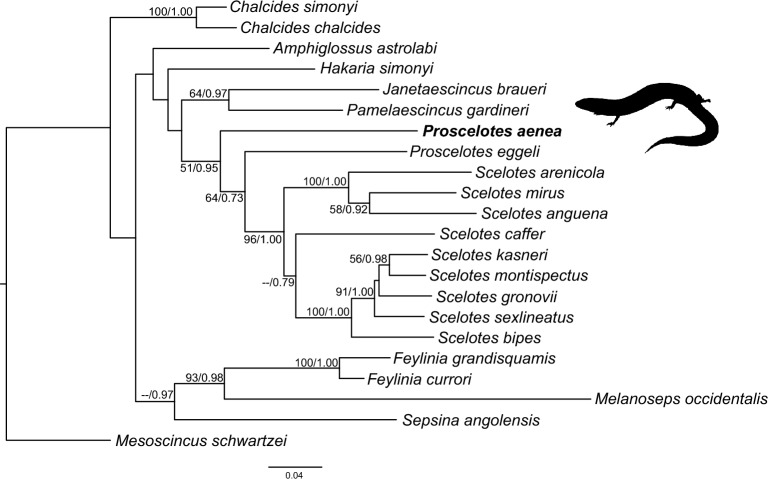
Phylogenetic position of *Proscelotes aenea*, inferred from concatenated 12S and 16S sequence data, inferred using maximum likelihood and Bayesian inference. The tree shown is a consensus tree based on the Bayesian analysis, and values on the branches indicate maximum likelihood bootstrap values > 50% and Bayesian posterior probabilities > 0.70 (ML/PP). The phylogeny is rooted following Bradley et al. (2005). Further information about the sequences used, including taxon information and GenBank accession numbers, is in the Supplementary Information, Table S1.

The combined 12S and 16S phylogeny indicates weakly (51% maximum likelihood bootstrap support, BS, and 0.95 Bayesian posterior probability, PP) that *Proscelotes* and *Scelotes* together make up a monophyletic group (Fig. 2). No strong support for the relationship between *P. aenea* and *P. eggeli* was found, but a paraphyletic relationship was weakly supported with *P.s aenea* sister to *P. eggeli* and *Scelotes* (64% BS/0.73 PP). Aside from this, the support for relationships among most other genera was poorly supported and often in conflict with earlier work. We expect this could be resolved further with the inclusion of more gene regions, but these relationships are beyond the scope of the current work and will not be discussed further.

## Discussion

Our one-year-long survey in Lumbo successfully located *P. aenea* and yielded novel information about the species, enabling us to better understand its biology, ecology and population status.

### Biology and ecology of *Proscelotes aenea*

Both oviparous and viviparous modes of reproduction are reported within the genus *Proscelotes*. Eggs (4–5) and embryos (5–6) have been found in different females of *P. arnoldi*^5^ and two embryos in a single *P. eggli* female^11^. There are no records on the biology or ecology of *P. aenea,* apart from two females taken at Lumbo in July 1918, each with two eggs measuring 7 × 5 mm^12^. Viviparity is already known within the genus^13^, and here we scientifically documented the first recorded birth of one *P. aenea* neonate. These findings suggest that the species' reproduction mode may either be ovoviviparous or both oviparous and viviparous, as is the case with *P. arnoldi*. The fact that oviparous species may be especially vulnerable under climate warming compared to sympatric viviparous species^14^ may explain the switch from oviparity to viviparity, however, more research is needed to confirm this assumption since it is based on just two individuals.

The reproductive period of the species seems to be between July and October. The evidence for this conclusion comes from the observation of *P. aenea* eggs in July^1^ and a pregnant individual in September in this study. Furthermore, all juveniles in this study were collected between September 27th and October 12th. The maximum individual size that we recorded is almost twice the length recorded in 1918. In that report, the maximum length of the males and females was 63.32 mm and 67.38 mm, respectively, but both individuals had short and regenerated tails. Our two largest specimens were 107 mm (62 mm SCL) and 110 mm (70 mm SCL) in length. The bright blue tail is also documented for the first time, as the specimens in 1918 were described as having plumbeous tails. We found that the tail was bright blue in males and juveniles.

We also document the species' ability to dig on the superficial layer of the soil with ease, a behaviour recorded previously for the genus^6^. We supply videos of the locomotion in the supplementary materials.

The genus *Proscelotes* is known to feed on different invertebrates such as the larval stages of various groups, as well as adults of beetles, cockroaches, and spiders^5^. Since the specimens fed exclusively on termites (*Cubitermes* sp. and *Macrotermes* sp.) and one male was also found inside a termite mound, it is likely that this species is a termite specialist.

### Distribution of *Proscelotes aenea*

In addition to the earlier records from Lumbo^12^, *P. aenea* was also recorded from Pemba, 220 km northwards of Lumbo, in 1948^2^. However, the species has not been recorded from Pemba since then, and a recent checklist that compiled 20 years of observations of and recorded 35 reptiles in Pemba failed to record the species^3^. Since *P. aenea* was last recorded in Pemba, the city’s population has grown from ca. 17,000 to ca. 200,000^15^, with extensive building, especially in sandy coastal areas where the species is known to occur. The apparent disappearance of *P. aenea* in Pemba and the decline observed in Lumbo may be linked to urbanization (1.1. Housing & urban areas under the IUCN Redlist threat criteria), which causes habitat loss due to changes in land and vegetation essential to the species' survival. This assumption is also valid for the species *Scolecoseps boulengeri*, also previously known from Pemba and Lumbo and collected in the same period as *P. aenea*. Unlike the latter, *S. boulengeri* was never found in Lumbo during this study, suggesting that the species has been extirpated from both Pemba and Lumbo. *Proscelotes aenea* is found in coastal vegetation on sandy soils in northern Mozambique and may be restricted by geographical barriers such as rivers or unsuitable hard soils. Due to the lack of broader surveys of herpetofauna in northern Mozambique^10^, it is not possible to exclude the possibility that the species occurs in the area between Pemba and Lumbo. This area is sparsely inhabited by people and has many habitats similar to the ones where individuals were found in this study, so it is possible that the species persists along the coast between the cities. Lumbo remains the only site where the species is unequivocally known to occur today. It is therefore urgent to sample the areas of suitable habitat and less urbanized coastal areas around Lumbo and Pemba, and elsewhere, to better understand the full distribution of the species.

### Phylogenetic position of *Proscelotes aenea*

We carried out a phylogenetic analysis using both the 12S and 16S regions to identify the evolutionary relationship among *P. aenea* and closely related taxa, as no genetic information was previously available for this species (Fig. 2). In line with earlier results^16,17^ we find that *Proscelotes* and *Scelotes* together make up a monophyletic group, although the support for this was low, 51% maximum likelihood bootstrap support (BS) and 0.95 Bayesian posterior probability (PP). The support for the relationship between the two species of *Proscelotes* was poorly resolved but a paraphyletic relationship was weakly supported with *P. aenea* sister to *P. eggeli* and *Scelotes* (64% BS/0.73 PP). While the results are not strong enough to make any firm conclusions, this may suggest that *Proscelotes* is not monophyletic. The addition of more markers, as well as sequence data for *P. arnoldi,* may further prove valuable in resolving the species-level relationships.

Unlike *Proscelotes,* the other genera with more than one species sampled have well supported congeneric species clusters (*Chalcides* and *Feylinia* both with 100% BS/1.00PP; *Scelotes* with 96% BS/1.00 PP). This suggests that irrespective of whether or not *Proscelotes* is monophyletic, *P. aenea* seems that it diverged earlier from *P. eggeli* than the taxa within other genera have from one another. This again suggests that *P. aenea* likely deserves a high priority in prioritization schemes incorporating phylogeny such as e.g. the EDGE of Existence program^18^.

Based on the species distribution, habitat and ecology, we propose changing the common name of *P. aenea* from Montane skink to Mozambique sand skink. This change is due to the current knowledge that the species is endemic to Mozambique and has only been reported from the coastal sand habitat and not mountains, unlike the other members of the genus.

### Conservation status of *Proscelotes aenea*

Between August and September of 1918, Loveridge recorded eight individuals of *P. aenea* while doing multi-taxa collections in the area for a period of two months. Since these specimens were collected while clearing land for tents, it is possible that more specimens were eventually observed and ignored. Here, using four different methods, especially targeting this species at five sites and for 365 consecutive days, we only found eight individuals. Although fossorial taxa are notoriously hard to find, we interpret these differences as putative evidence for a decline in the abundance of the species in Lumbo. This, as well as the probable extirpation of the species from Pemba^3^, suggests that urbanization plays a role in the decline of the species.

The International Union of the Conservation of Nature (IUCN) Red List assessment currently reports *P. aenea* as Data Deficient. Based on the information presented here, and based on the evidence of its disappearance from Pemba^3^, we can with some degree of confidence infer a steep population decline of the species in Lumbo. The number of confirmed locations of the species also decreased from two to one since it is now only known to occur in Lumbo. Surveys are therefore urgently needed in less urbanized areas between Pemba and Lumbo and south of Lumbo to map the extant distribution of the species. If such surveys fail to record the presence of the species, it is likely that this species is under threat and should be placed in a threatened IUCN category on the basis of its range and observed decline.

## Conclusions and recommendations

Primary data on biodiversity is crucial to sustaining evidence-based conservation decisions^19^. Biodiversity data is however severely biased in terms of spatial distribution^10^, taxonomic groups^20^, countries^21^ and accessibility^22^. One additional bias that is rarely mentioned is the conspicuous nature of some organism groups^23^. Species occur at varying relative abundance^24^ and the ones occurring at lower densities may be especially hard to document. As we show here, finding rare species can be a true challenge and relying on the scarcity of international expeditions is not a viable strategy—as they tend to be expensive and short-term.

We therefore recommend that local universities and research centres take the lead on the documentation of biodiversity and address the lack of studies that can thoroughly document regional and local biodiversity, including seldomly seen and poorly known species. For some taxonomic groups, such as the rare skink we researched here, this can only be done by implementing intensive sampling campaigns using very specific and targeted methods. Besides producing crucial baseline information on national biodiversity, such work helps strengthen the local scientific and conservation capacity of students and professionals, and to engage communities which are the long-term stewards of land and ecosystems. To deliver on the zero-extinction commitments set by the Kunming-Montreal Global Biodiversity Framework, urgent investments are needed in economically deprived, but species-rich regions such as much of the tropics.

## Methods

### Study area and site description

**Figure 3:**
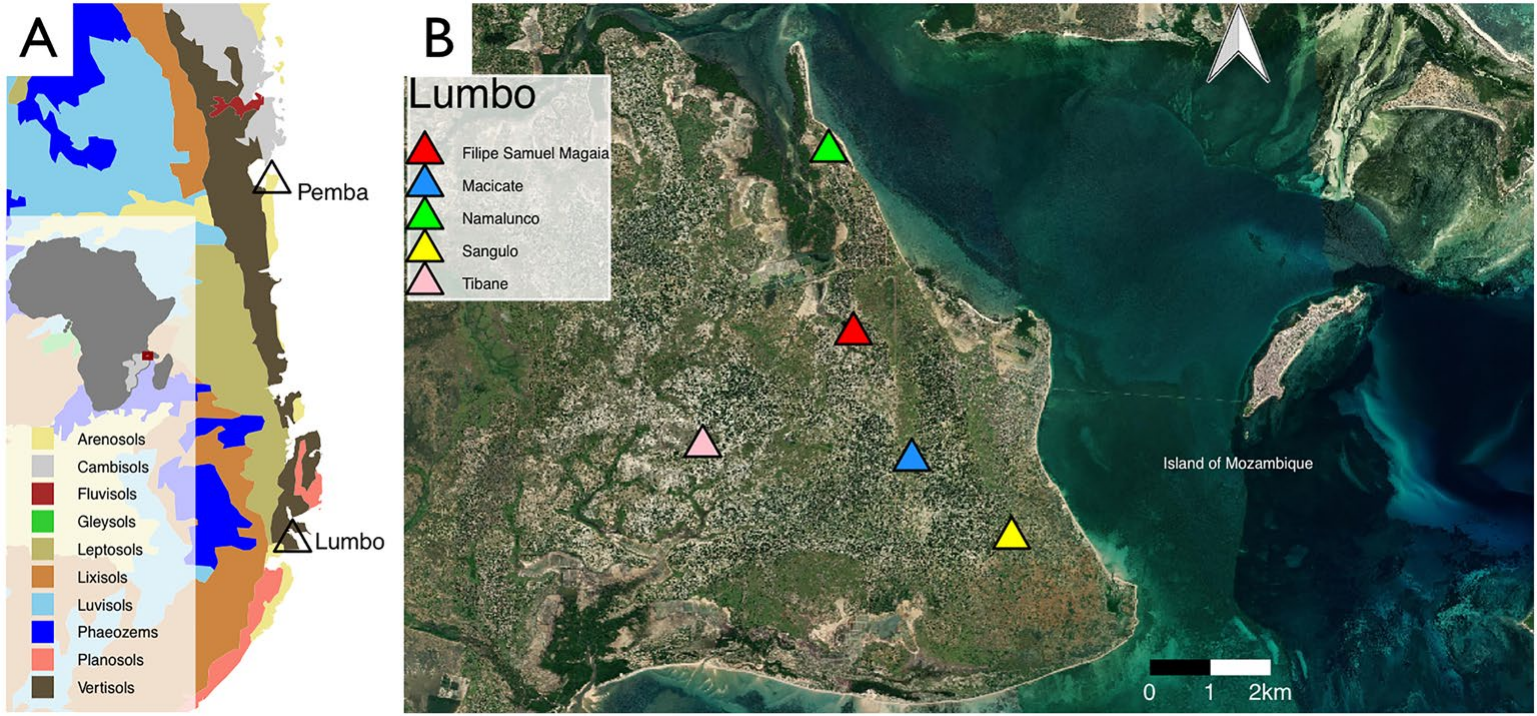
(**A**) Major soil types^28^ in and around Pemba and Lumbo. (**B**) Sampling sites for this study. *P. aenea* was found at Filipe Samuel Magaia, Namalunco and Tibane. Sattelite imagery obtained from Bing^29^, and plotted in R (*version* 4.1.2)^30^.

We placed five trapping systems at different sites in and around Lumbo, Mozambique (Fig. 3). Lumbo is an administrative post in the district of Ilha de Moçambique and a moderately urbanized area with a population of 56,648 recorded in 2017 [INE,^25^]. The population has almost doubled since the previous census in 2007 when it was 31,483 [INE,^26^]. In terms of available habitats, the landscape is now mostly transformed due to agricultural activity and housing. Prior to the expansion of the human settlement, the vegetation cover in Lumbo was a coastal thicket, but most of the trees are now either mango or cashew, and few patches of the original vegetation remain. The climate is warm and humid, with a dry winter. The wet season occurs between November and May and the dry season is between June and October. The precipitation shifts from a monthly average of 220 mm in the wet season to less than 35 mm in the dry season. The temperature however remains constant throughout the year, with an average of 25.5 °C and less than 2.5 °C of variation between the monthly averages [MICOA,^27^].

**Figure 4:**
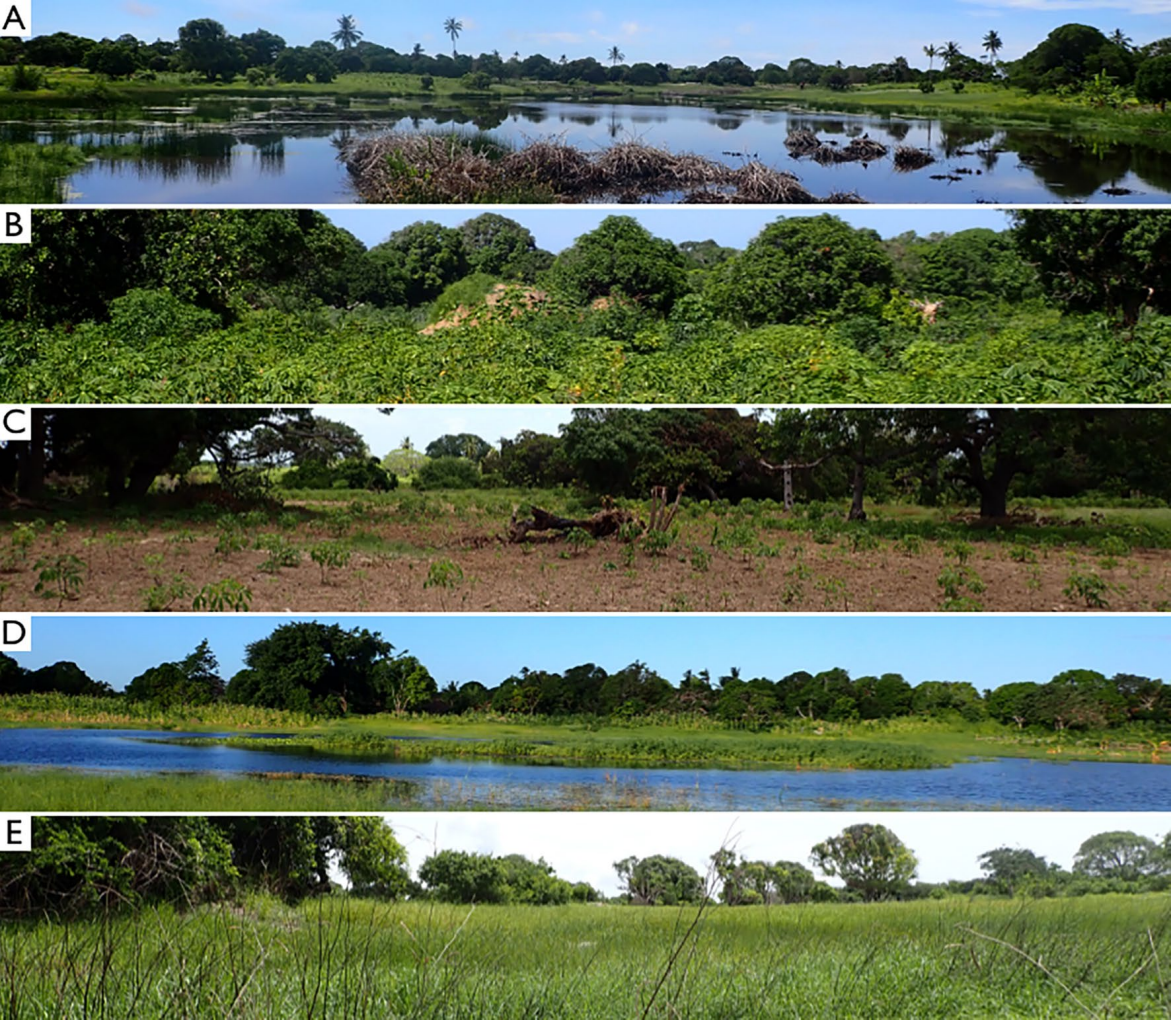
Habitats of the sampling sites: (**A**) Filipe Samuel Magaia, (**B**) Mancicate, (**C**) Namalunco, (**D**) Sangulo, (**E**) Tibane. Numalunco and Macicate had higher degrees of habitat degradation compared to the other sites.

The sampling was conducted at five sites near Lumbo (Figs. 3, 4): (1) Filipe Samuel Magaia (40.66531°, − 15.03501°, 12 m a.s.l), (2) Mancicate (40.67437°, − 15.05459°, 14 m a.s.l), (3) Namalunco (40.66149°, − 15.00670°, 12 m a.s.l), (4) Sangulo (40.68983°, − 15.06670°, 9 m a.s.l), (5) Tibane (40.64194°, − 15.05245°, 22 m a.s.l), based on the presence of water bodies, vegetation type, elevation, and soil type, in order to cover a diversity of habitats.

The first four sites (Filipe Samuel Magaia, Macicate, Sangulo and Tibane) were selected to cover a diversity of habitats. A fifth site was added (Namalunco) on August 6th (on the 116th day of sampling) based on the description of the campsite in Arthur Loveridge’s biography^31^ where *P. aenea* was previously found. All sites had some degree of urbanization and land transformation, but Tibane stands out as the least disturbed site, with the most pristine vegetation cover and native herbaceous plants. Conversely, Namalunco and Macicate are the sites with the highest degrees of disturbance and urbanization. Filipe Samuel Magaia and Sangulo had moderate degrees of anthropogenic disturbance. Habitat features such as wetlands and termite mounds were present at every site except Namalunco (Fig. 3).

### Sampling methods

Sampling commenced on April 12, 2021, continued for 365 uninterrupted days until April 12, 2022, and consisted of four sampling methods: (1) active search, (2) pitfall traps with drift fences, (3) funnel traps placed along the drift fences and (4) provision of artificial refugia.

Each of the five trapping systems consisted of three sunken plastic buckets of 25 L (L) each, serving as pitfall traps, respectively placed close to each end and in the middle of a sunken plastic drift fence (10 m long × 0.4 m high). Along each drift fence, we placed one two-way funnel trap (https://traps.com.au/product/funnel-trap/) on either side and two homemade one-way funnel traps, positioned respectively at each end of the drift fence (see FigS1-2). The artificial refugia consisted of three plywood board (1.20 m by 0.5 m) placed flat on top of the soil (Fig. S1-2). Pitfalls, funnels and artificial refugia were inspected every morning. Active search was conducted every day in the morning for a period of 1–2 h between 6:00 am and 12:00 pm. Photos of the collection methods are provided in the supplementary materials (SM1, FigS1-2).

### Collection and preservation

We collected eight specimens of *P. aenea*, over a period of a year—two individuals every three months. Specimens were euthanized with MS222 following the recommendations of ethical committees for animal experimentation^32^ and fixed in formalin for 15 days before being transferred to ethanol 70%. All specimens were photographed in life prior to euthanasia and measured after euthanasia (STL, STC and TL) and prior to preservation. Tissue samples were taken from each individual and stored in 99% ethanol. The voucher specimens were deposited at Lúrio University and registered with GBIF (https://doi.org/10.15468/dl.8kydfs).

### Biology and ecology

To record information on the biology and ecology of the species we kept four *P. aenea* individuals in captivity (Catalogue numbers at the Faculty of Natural Sciences: EOS144, EOS145, EOS173 and EOS175) and provided each with a variety of food sources—spiders, ants, earthworms, hemipterans, and termites. The specimens only preyed on termites and abstained from eating the other invertebrates even if termites were not available. One individual (EOS173) gave birth in captivity to the individual (EOS175). The birth took place underground.

### Molecular analysis

DNA was extracted from the preserved liver samples of *P. aenea* specimens EOS144, EOS145, and EOS157 using the Qiagen DNeasy Blood and Tissue kit following the manufacturer’s protocol^33^. Portions of the mitochondrial genes 12S, 16S, and ND2 were amplified and sequenced using the PCR primers and PCR thermocycler protocols following Heinicke, Turk^34^ (Table 2). Following the PCRs, DNA was purified and sequenced at the Eurofins DNA sequencing core. The ND2 sequences were of relatively poor quality and phylogenetic analyses were therefore restricted to 12S and 16S genes. The novel sequences were deposited in NCBI GenBank and are openly accessible (See Data availability statement).

**Table 2:** PCR primers and thermocycling programs used to amplify the target sequences: 12S, 16S, and ND2.

Genes	Primers	PCR cycles
12S	12SA-L	94 °C 180 s, [(94 °C 60 s, 50 °C 60 s, 72 °C 60 s) × 40], 72 °C 300 s
	12SB-H	
16S	16SL (16sar-L Palumbi 1991)	94 °C–90 s, [(94 °C–45 s, 55 °C–45 s, 72 °C–90 s) × 33], 72 °C–600 s
	16SH (16sbr-H Palumbi et al. 1991)	
ND2	H5617a	94 °C 90 s (94 °C 30 s, 45 °C 45 s, 72 °C 90 s) X 35 72 °C 600 s
	L4882	

Sequences were edited and assembled in Geneious Prime (Version 2022.2.2). To infer the phylogenetic relationships, 12S and 16S sequences from closely related species were downloaded from GenBank (Table S1, Supplementary Information). Sequences from *Proscelotes*, *Scelotes,* and *Sepsina* were included, as well as sequences from three of the four other mainland African genera (*Chalcides*, *Feylinia*, and *Melanoseps*), following earlier phylogenetic findings^16^. No 12S or 16S sequences were available from the genus *Scolecoseps*. In addition, we included a selection of species from the clades found within Indian Ocean Island radiations (mainly Madagascar but also Socotra and Mascarenes). *Mesoscincus schwartzei,* was used as an outgroup. The phylogeny included a total of 21 species with both 12S and 16S sequences. In some cases, 12S and 16S sequence data used in the concatenated tree come from different individuals of the same species. No incongruencies were found in the single gene trees prior to concatenation. Supplementary Table S1 lists all species names, voucher IDs where available and GenBank accession numbers for the taxa used in phylogenetic reconstruction.

Phylogenies were constructed using standard maximum likelihood and Bayesian approaches. Sequences were aligned in AliView Version 1.28^35^. The Bayesian phylogenies were inferred using MrBayes 3.2. (https://www.ncbi.nlm.nih.gov/pmc/articles/PMC3329765/) with separate partitions for 12S and 16S at GTR + gamma model. Small parts of the sequences could not be reliably aligned due to indels and these were masked from the alignment. The models ran for 5 million generations with 1,250,000 generations as burn-in. Single gene trees were inferred and visually inspected for well-supported incongruencies (> 0.70 Bayesian posterior probability) before the two datasets were concatenated. The maximum likelihood phylogenies were inferred using raxmlGUI 2.0^36^ with the ML + thorough bootstrap + consensus settings, 100 runs and 1000 replicates, and a GTR substitution model. As with the Bayesian analyses, the two regions were partitioned, and the same indel-heavy regions were excluded from the analyses.

## Supplementary Information

Supplementary Information 1.

Supplementary Information 2.

## Data Availability

The datasets generated and/or analyzed during the current study are available in the GenBank and GBIF repositories. https://submit.ncbi.nlm.nih.gov/subs/genbank/SUB12872088, https://submit.ncbi.nlm.nih.gov/subs/genbank/SUB12843287, https://submit.ncbi.nlm.nih.gov/subs/genbank/SUB12843061, https://submit.ncbi.nlm.nih.gov/subs/genbank/SUB12843158, https://submit.ncbi.nlm.nih.gov/subs/genbank/SUB13084977, https://submit.ncbi.nlm.nih.gov/subs/genbank/SUB13084980,. GBIF (https://doi.org/10.15468/dl.8kydfs). photos and videos of the species are freely available on the project’s website: www.extinctorshy.org and supplementary material.
