# Supplementary material for: Rediscovery of the lost skink Proscelotes aenea and implications for conservation
Source: Sci Rep. 2023 Jul 12;13:11261. doi: 10.1038/s41598-023-38286-4 (PMC10338472; doi:10.1038/s41598-023-38286-4)
Supplement: Supplementary file 1 — Supplementary Information 1. [file 41598_2023_38286_MOESM1_ESM.docx]

**Supplementary information**

Table S1. Taxa and NCBI GenBank accession numbers for the taxa used to infer the phylogenies in Figure 4.

| **Taxon** | **Voucher ID** | **12S Accession** | **16S Accession** |
| --- | --- | --- | --- |
| *Proscelotes aenea* | EOS144 | SUB13084977 | *SUB12872088* |
| *Proscelotes aenea* | EOS145 | SUB13084980 | *SUB12843158* |
| *Proscelotes aenea* | EOS157 | NA | *SUB12843061* |
| *Melanoseps occidentalis* | S11 | NA | AY217973 |
| *Feylinia grandisquamis* | F1 | AY218002 | AY217952 |
| *Feylinia currori* | E62 | AY308369 | AY308220 |
| *Mesoscincus schwartzei* | JAC_21060 | AY649117 | AY649158 |
| *Janetaescincus braueri* | - | AY649123 | AY649164 |
| *Amphiglossus astrolabi* | - | AY391125 | AY391143 |
| *Chalcides simonyi* | E3007.2 | EU277872 | EU278030 |
| *Sepsina angolensis* | S13 | AY218024 | AY217975 |
| *Scelotes arenicola* | S7 | AY218038 | AY217988 |
| *Proscelotes eggeli* | S22 | AY218032 | AY217982 |
| *Proscelotes eggeli* | - | NA | AY315561 |
| *Chalcides chalcides* | - | AJ416936 | AJ416935 |
| *Hakaria simonyi* | - | AY649122 | AY649163 |
| *Scelotes mirus* | - | AY649130 | AY649171 |
| *Scelotes anguina* | S20 | AY218030 | AY217981 |
| *Scelotes anguena* | - | NA | AY028891 |
| *Pamelaescincus gardineri* | Chelex1 | AY308437 | AY308285 |
| *Scelotes caffer* | S4 | AY218035 | AY217985 |
| *Scelotes kasneri* | S6 | AY218037 | AY217987 |
| *Scelotes sexlineatus* | S2 | AY218033 | AY217983 |
| *Scelotes gronovii* | S5 | AY218036 | AY217986 |
| *Scelotes montispectus* | S17 | AY218027 | AY217978 |
| *Scelotes bipes* | S18 | AY218028 | AY217979 |


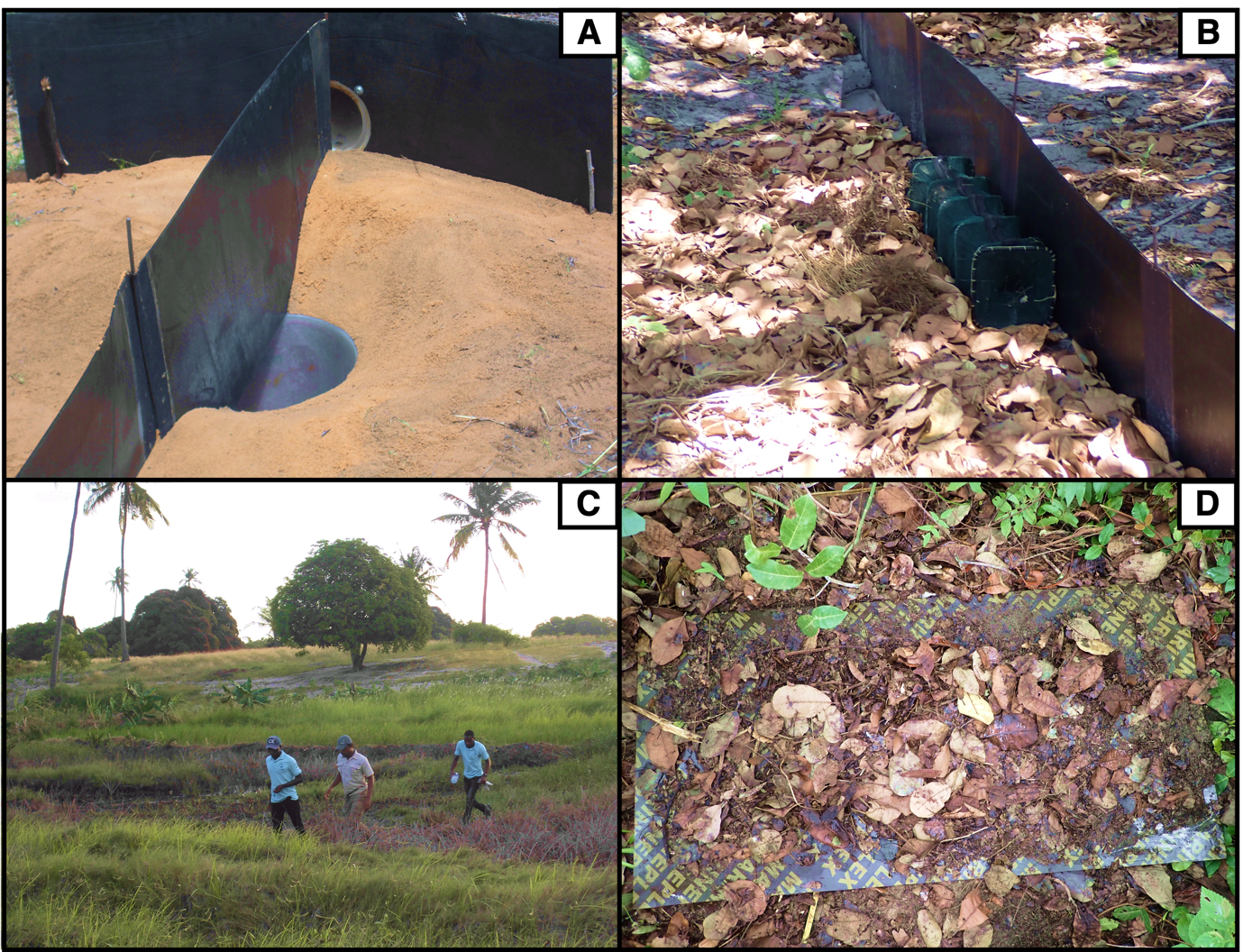


Figure S1: Photographs illustrating the various sampling methods used in the study. A. Pitfall traps with drift fencing, B. Funnel traps, C. Active search (from left to right: A. Jamal, C. Nanvonamuquitxo and W. Monia), D. Artificial refugia.


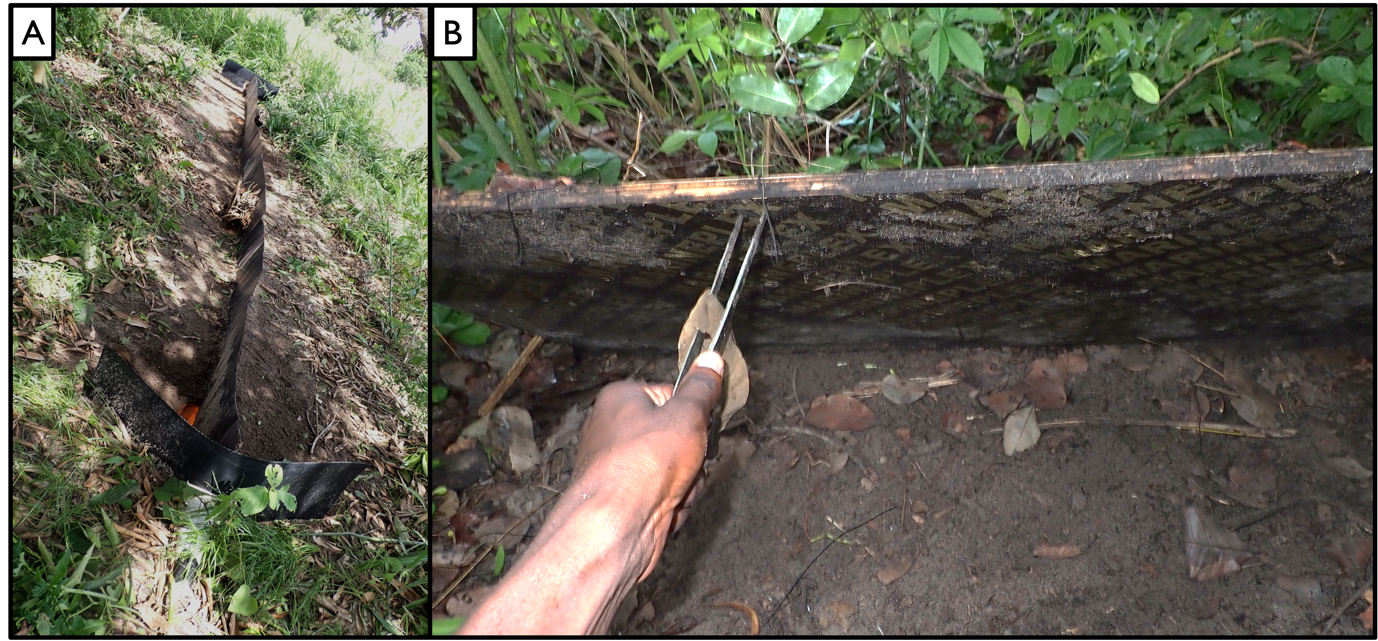


Figure S2: Additional photographs showcasing the trapping system and the thickness of the plywood sheets.


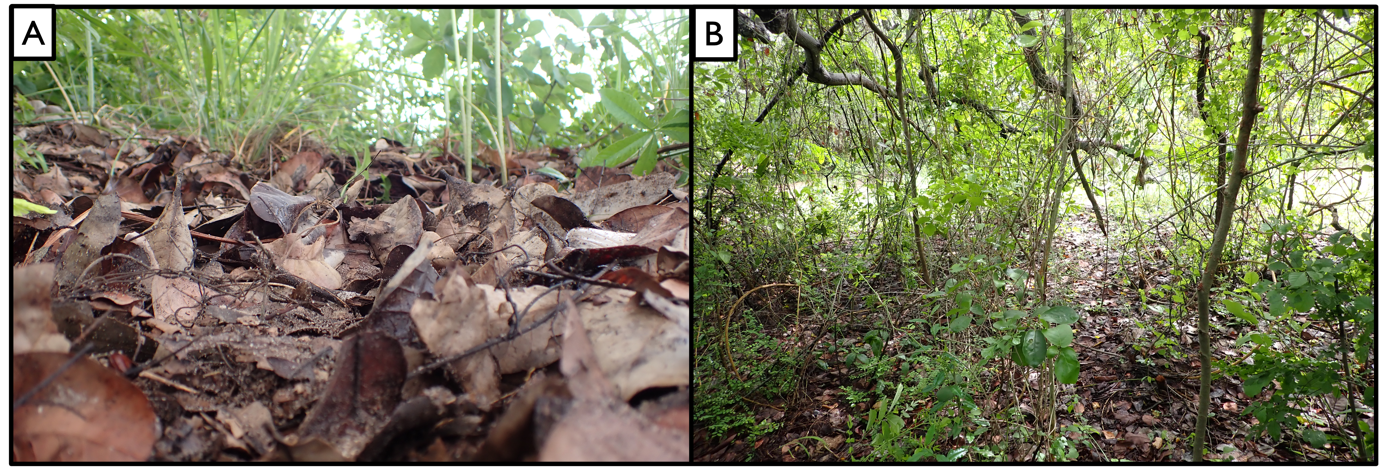


Figure S3: Additional photographs showcasing the habitats where the *P. aenea* specimens were collected.

Table S2: Comparison of 15 anatomical structures between the colected P. aenea specimens in this study and the ones in 1918.

|  | Puruleia et al. 2023 | Barbour and Loveridge, 1928 |
| --- | --- | --- |
| Toes | Five toes, two of them longer (third and fourth) than double the lengt of the others. | Five toes, two of them longer (third and fourth) than double the length of the others. |
| Snout | Slightly conical and projected downwards. | Slightly conical and projected downwards. |
| Supranasal | Two | Two |
| Frontonasal | One | One |
| Frontal | One, and double the length of the frontonasal. | One, and double the length of the frontonasal |
| Prefrontal | Absent | Absent |
| Infralabials | Six | Not mentioned on the description |
| Supralabials | Six, with the fifth touching the eye. | Six, with the fifth touching the eye. |
| Supraocular | Four | Four |
| Preocular | Two | Two |
| Postocular | One | One |
| Frontoparietal | Absent | Absent |
| Interparietal | One with an triangular shape and with a pineal eye. | One with an angular shape |
| Parietal | One | Not mentioned on the description |
| Supraciliary | Six | Six |
